# Atopische Dermatitis und Diabetes mellitus – Gibt es Zusammenhänge?

**DOI:** 10.1007/s00105-024-05440-6

**Published:** 2024-12-09

**Authors:** Adelina-Maria Sendrea, Carmen Maria Salavastru

**Affiliations:** 1 Carol Davila Universität für Medizin und Pharmacie, 8 Eroilor Sanitari Boulevard, 050474 Bukarest, Rumänien; 2 Abteilung Pädiatrische Dermatologie, Colentina Klinik, Bukarest, Rumänien; 3 Dermatologische Forschungsabteilung, Colentina Klinik, Bukarest, Rumänien

**Keywords:** Korrelation, Th1/Th2-Paradigma, Leptin, Adiponektin, Multidisziplinärer Ansatz, Correlations, Th1/Th2 paradigm, Leptin, Adiponectin, Multidisciplinary approach

## Abstract

**Hintergrund:**

Atopische Dermatitis und Diabetes mellitus sind chronische, immunvermittelte, entzündliche Erkrankungen, die die Lebensqualität der Patienten erheblich beeinträchtigen und zudem eine beträchtliche sozioökonomische Belastung darstellen. Trotz intensiver Forschung in den letzten Jahrzehnten bleibt der mögliche Zusammenhang zwischen diesen beiden medizinischen Zuständen aufgrund spärlicher und manchmal widersprüchlicher Daten ein umstrittenes Thema. Dennoch beruht die potenzielle Verbindung zwischen ihnen auf einigen anerkannten gemeinsamen pathophysiologischen Merkmalen.

**Ziele:**

Ein möglicher Zusammenhang zwischen atopischer Dermatitis und Diabetes mellitus soll dargestellt und bewertet werden.

**Material und Methoden:**

Wir führten eine Literaturrecherche zum potenziellen Zusammenhang zwischen atopischer Dermatitis und Diabetes mellitus durch.

**Ergebnisse:**

Mehrere Studien haben eine Korrelation zwischen atopischer Dermatitis und Diabetes mellitus Typ 1 oder Typ 2 festgestellt. Andere Studien zeigten jedoch keinen Zusammenhang zwischen diesen beiden Erkrankungen oder deuteten sogar darauf hin, dass atopische Dermatitis das Risiko für die Entwicklung von Diabetes mellitus bei bestimmten Patienten verringern könnte. Darüber hinaus weisen diese beiden chronischen Erkrankungen auch bestimmte klinische Merkmale auf, die auf eine mögliche Korrelation hindeuten. Derzeit gibt es jedoch keinen eindeutigen wissenschaftlichen Beweis für einen signifikant positiven Zusammenhang zwischen atopischer Dermatitis und Diabetes mellitus, was v. a. auf das Fehlen umfangreicher und vielfältiger demografischer Studien zurückzuführen ist.

**Schlussfolgerungen:**

Ärzte sollten sich dieser potenziellen Korrelation sowohl bei Erwachsenen als auch bei pädiatrischen Patienten bewusst sein und die Bedeutung eines multidisziplinären Ansatzes für das Management der atopischen Dermatitis berücksichtigen. Weitere Untersuchungen sind erforderlich, um mögliche Zusammenhänge zwischen atopischer Dermatitis und Diabetes mellitus in spezifischen Bevölkerungsgruppen zu bestimmen.

## Abstract (en)

**Background:**

Atopic dermatitis and diabetes mellitus are chronic, immune-mediated, inflammatory diseases that significantly affect patients’ quality of life and also represent a considerable socioeconomic burden. Despite intensive research in recent decades, the possible link between these two medical conditions remains a controversial topic due to sparse and sometimes contradictory data. Nevertheless, the potential link between them is based on some recognized common pathophysiological features.

**Aim:**

To summarize and evaluate a possible association between atopic dermatitis and diabetes mellitus.

**Materials and methods:**

A literature review on the potential association between atopic dermatitis and diabetes mellitus was conducted.

**Results:**

Several studies have reported a correlation between atopic dermatitis and type 1 or type 2 diabetes mellitus. However, other studies have shown no association between these two conditions or even suggested that atopic dermatitis could reduce the risk of developing diabetes mellitus in certain patients. In addition, these two chronic diseases also have certain clinical features that suggest a possible correlation. However, there is currently no clear scientific evidence of a significant positive association between atopic dermatitis and diabetes mellitus, mainly due to the lack of large and diverse demographic studies.

**Conclusion:**

Clinicians should be aware of this potential correlation in both adult and pediatric patients and consider the importance of an interdisciplinary approach for the management of atopic dermatitis. Further research is needed to determine possible associations between atopic dermatitis and diabetes mellitus in specific populations.

Atopische Dermatitis und Diabetes mellitus sind komplexe entzündliche Erkrankungen, die durch immunologische Dysregulation und Umweltfaktoren bei genetisch prädisponierten Personen beeinflusst werden und weltweit zunehmend verbreitet sind. Atopische Dermatitis, die ursprünglich nur als Hauterkrankung betrachtet wurde, wird inzwischen als systemische Erkrankung erkannt, die möglicherweise Verbindungen über die Haut hinaus aufweist, einschließlich Diabetes mellitus. Der Zusammenhang zwischen diesen beiden Erkrankungen ist aufgrund widersprüchlicher Daten in der Literatur weiterhin umstritten.

## Background

Die atopische Dermatitis (AD) ist eine chronische, entzündliche Hauterkrankung, die überwiegend Kinder (bis zu 20 %) betrifft, jedoch auch in geringerem Maße Erwachsene [1]. Ihre Entwicklung, obwohl noch nicht vollständig verstanden, wird durch eine komplexe Interaktion von Faktoren bestimmt, einschließlich Hautbarrieredefekten, genetischer Prädisposition, dauerhaftem entzündlichen Milieu, Haut- und Darmdysbiose sowie Th-gesteuerten immunologischen Reaktionen [2]. Die Diagnose der atopischen Dermatitis basiert auf der klinischen Untersuchung, da sie sich durch wiederkehrende, juckende Schübe manifestiert, die durch Ekzemläsionen gekennzeichnet sind und in Morphologie und Lokalisation je nach Alter des Patienten variieren. Abgesehen von der Hautbeteiligung können Personen mit atopischer Dermatitis zusätzliche klinische Manifestationen aufweisen, die auf eine systemische Beteiligung hindeuten. Das Konzept des „atopischen Marsches“ umfasst die bekanntesten und anerkanntesten extrakutanen Erkrankungen, die Patienten mit atopischer Dermatitis begleiten können, nämlich die sequenzielle Entwicklung allergischer Erkrankungen, beginnend mit Nahrungsmittelallergien im Säuglingsalter und fortschreitend zu allergischer Rhinitis und Asthma im Kindesalter [3]. Abgesehen von diesem Konzept unterstützen immer mehr Studien die Auffassung, dass atopische Dermatitis eine systemische Erkrankung ist, die mit verschiedenen potenziellen Komorbiditäten einhergeht, darunter möglicherweise Insulinresistenz oder Diabetes mellitus [4, 5].

Diabetes mellitus (DM) ist eine chronische, komplexe Stoffwechselerkrankung, die durch Hyperglykämie gekennzeichnet ist und durch eine Störung des Glukosestoffwechsels verursacht wird. Diese führt zu einem Insulinsekretionsmangel (absolut oder relativ), einer peripheren Resistenz gegenüber den Effekten von Insulin oder einer Kombination beider Mechanismen [6]. Klinisch äußert sich DM in einer Vielzahl von Symptomen, die verschiedene Systeme betreffen können, wie Polyurie, Polyphagie, Polydipsie sowie Gewichtsverlust, Müdigkeit und Reizbarkeit [7]. Schlecht kontrollierter DM kann zu verschiedenen Komplikationen führen. Die Pathogenese beruht je nach Typ entweder auf einer autoimmunen Zerstörung der Pankreaszellen durch T‑Zellen, die zu einem absoluten Insulinmangel führt (Typ-1-DM), oder auf einer genetisch-umweltbedingten Interaktion (Typ-2-DM) [6].

## Are atopic dermatitis and diabetes mellitus interconnected?

### Klinische Merkmale

Hauttrockenheit (Xerose), Juckreiz (Pruritus) und Keratosis pilaris sind häufige klinische Merkmale, die bei Patienten mit AD oder DM auftreten. Pruritus, das Leitsymptom bei AD, betrifft bis die Hälfte der Patienten mit DM und steht in engem Zusammenhang mit Hautxerose, die bei beiden Erkrankungen teilweise ein Ichthyose-ähnliches Erscheinungsbild annehmen kann [8, 9].

### Pathophysiologie

Die mögliche Verbindung zwischen AD und DM basiert weitgehend auf bestimmten gemeinsamen pathophysiologischen Mechanismen einschließlich eines anhaltenden proinflammatorischen Zustands und immunologischer Reaktionen sowie epithelialer Dysfunktion, Umweltfaktoren und genetischer Veranlagung.

### Entzündung und immunologische Reaktionen

Obwohl AD primär eine durch Th2 getriebene Entzündungskrankheit ist, während Typ-1-DM hauptsächlich durch Th1-Reaktionen beeinflusst wird, können bestimmte proinflammatorische Zytokine, die bei der atopischen Dermatitis beteiligt sind, ebenfalls zum entzündlichen Milieu beitragen, das bei DM beobachtet wird. Folglich wird das ursprünglich entwickelte Th1/Th2-Paradigma, das postulierte, dass sich diese beiden Krankheiten gegenseitig ausschließen, nun als zu vereinfachend betrachtet. Es entstand ein neues, überarbeitetes Th1/Th2-Paradigma, das darauf hinweist, dass Th1- und Th2-getriebene Krankheiten nebeneinander bestehen können [10]. Einige Adipokine (d. h. aus Fettgewebe stammende Zytokine) sind als gemeinsame Nenner sowohl bei AD als auch bei DM zu finden. Erhöhte Leptinwerte (ein proinflammatorisches Adipokin) und verringerte Adiponektinwerte (ein antiinflammatorisches Adipokin) wurden bei Patienten mit AD [11] und Typ-2-DM [12] festgestellt.

Die Hygienehypothese und die Dysfunktion der epithelialen Barriere, Schlüsselkomponenten bei der Entstehung von AD [13], spielen nachweislich auch bei DM eine Rolle, hauptsächlich bei Typ 1, aber auch bei Typ 2 [14, 15], ebenso wie genetische Faktoren, die ein gemeinsamer Nenner für AD und Typ-1-DM sind.

### Gemeinsame Komorbiditäten und Risikofaktoren

Ein wenig bewegungsaktiver Lebensstil wird sowohl bei AD als auch bei DM beschrieben. Erwachsene mit AD neigen zu geringerer körperlicher Aktivität [16], während AD-diagnostizierte Jugendliche eine verringerte maximale Leistungsfähigkeit und niedrigere wöchentliche Trainingswerte aufweisen und ihnen im Vergleich zu Kontrollpersonen ohne AD das Selbstvertrauen fehlt, eigenständig regelmäßige körperliche Aktivitäten zu planen [17]. Mehrere Faktoren können diese Befunde bei AD-Patienten erklären (Schlafstörungen, Ekzemläsionen, Hyperhidrose und erhöhte Körpertemperatur während des Trainings als Auslöser für Ekzeme) [5, 16]. Bei Typ-2-DM ist bekannt, dass ein höheres Aktivitätsniveau mit einem reduzierten Diabetesrisiko verbunden ist, da ein körperlich inaktiver Lebensstil Übergewicht, eine eingeschränkte Blutzuckerkontrolle und Insulinresistenz fördert [18].

Wenig bewegungsaktiver Lebensstil und Fettleibigkeit sind eine gemeinsame Komorbidität für AD und DM

Fettleibigkeit ist eine weitere gemeinsame Komorbidität für AD und DM. Während der Zusammenhang zwischen DM und Fettleibigkeit gut belegt ist (Adipositas im Kindes- und Jugendalter ist mit einer höheren Wahrscheinlichkeit verbunden, im Erwachsenenalter Typ-2-DM zu entwickeln [19], und ein höheres Körpergewicht bei jüngeren Menschen erhöht das Risiko für die Entwicklung von Typ-1-DM [20]), ist die Verbindung zwischen AD und Fettleibigkeit weniger eindeutig. Einige Studien identifizieren ein erhöhtes Risiko für Übergewicht und Fettleibigkeit bei AD-Kindern mit einem direkten Zusammenhang zur Schwere der AD [21], während andere diesen Zusammenhang unabhängig vom Alter des Patienten erwähnen [22]. Diese Befunde sind jedoch nicht durchgängig in der Literatur zu finden.

### Kardiovaskuläre Ereignisse

Sie treten häufig bei Patienten mit DM auf, da diese oftmals arterielle Hypertonie, Myokardinfarkt und Schlaganfall aufweisen [23]. Bei AD-Patienten ist der Zusammenhang mit kardiovaskulären Erkrankungen weniger gut belegt. Dennoch haben mehrere Beobachtungsstudien auf einen potenziellen Zusammenhang zwischen AD und einem erhöhten Risiko für die Entwicklung kardiovaskulärer Erkrankungen wie Myokardinfarkt, Herzinsuffizienz und Schlaganfall hingewiesen, möglicherweise in Verbindung mit der Schwere der AD [24].

## Welche Fakten sind bekannt?

Angesichts der potenziellen, aber nicht vollständig bewiesenen Verbindung zwischen AD und DM sowie der gemeinsamen klinischen und pathophysiologischen Merkmale und Komorbiditäten sind die Daten in der Literatur zu ihrem Zusammenhang umstritten und derzeit in Diskussion.

Die Tab. 1 listet einige der bisher in der Literatur verfügbaren und relevanten Studien auf.

**Tab. 1:** Zusammenfassung von Literaturdaten zur Verbindung von atopischer Dermatitis (AD) und Diabetes mellitus (DM)

Autoren	Jahr	Forschungsart	Altersgruppe der Bevölkerung	Schlussfolgerungen
*Pro AD-DM-Verbindungen*				
Lin CH et al. [25]	2016	Retrospektive Fall-Kontroll-Studie	Kinder (Durchschnittsalter 10,4 Jahre, respektive 11,6 Jahre)	Höhere Inzidenzrate von AD in der Typ-1-DM-Kohorte, selbst nach Anpassung für Risikofaktoren
Silverberg J et al. [26]	2018	Querschnittsstudie	Erwachsene	Höhere Wahrscheinlichkeit bei AD-Patienten für selbst berichtete Komorbiditäten (DM, Adipositas, arterielle Hypertonie, Herzkrankheit), die direkt proportional zur Schwere der AD sind
*Kontra AD-DM-Verbindungen*				
Lu F et al. [27]	2023	Mendelsche Randomisierungsstudie	–	Genetisch vorhergesagte AD ist positiv mit einem erhöhten Risiko verbunden, DM (Typ 1 und Typ 2) zu entwickeln
Gazit V et. al. [28]	2008	Kontrollierte Fallstudien	Kinder und Jugendliche	Kein Unterschied in der Prävalenz von Atopie (AD, Serum-IgE-Spiegel, Empfindlichkeit gegenüber luftgetragenen Allergenen) bei insulinabhängigen Patienten im Vergleich zu Kontrollen
EURODIAB study group [29]	2000	Multizentrische Fall-Kontroll-Studie	Kinder	Geringere Häufigkeit atopischer Erkrankungen bei Kindern, die in Westeuropa mit Typ-1-DM diagnostiziert wurden
Drucker et al. [30]	2017	Querschnittsstudie	Erwachsene	Keine Assoziation zwischen AD und Typ-2-DM; AD hat schützende Effekte gegen die Entwicklung von Typ-2-DM

*IgE* Immunglobulin E

## Ausblick

Weitere Forschungen, die große und unterschiedliche Bevölkerungsgruppen (in Bezug auf Ethnie, Alter und geografische Lage) einbeziehen, sind erforderlich, um das gleichzeitige Auftreten von atopischer Dermatitis und Diabetes mellitus besser zu verstehen. Darüber hinaus sollten zukünftige molekulare Studien die potenzielle Rolle von Th1-vermitteltem Diabetes mellitus bei der Entwicklung und dem Fortschreiten von Th2-gesteuerter atopischer Dermatitis und umgekehrt untersuchen.

Solche neuen Forschungsansätze könnten prognostische und therapeutische Implikationen haben, wie z. B. eine frühzeitige Diagnose und Behandlung der atopischen Dermatitis, die potenziell das Risiko der Entwicklung von Typ-1- und Typ-2-Diabetes senken könnten.

## Fazit für die Praxis

- Auch wenn es keine definitiven Beweise gibt, die einen stark positiven Zusammenhang zwischen atopischer Dermatitis und Diabetes mellitus bestätigen, ist es für Gesundheitsdienstleister, einschließlich Dermatologen, Kinderärzte, Endokrinologen und Ernährungsspezialisten, wichtig, sich des potenziellen Zusammenhangs zwischen atopischer Dermatitis und Diabetes mellitus bei Erwachsenen und Kindern bewusst zu sein.
- Die multidisziplinäre Zusammenarbeit spielt eine entscheidende Rolle im effektiven Management der atopischen Dermatitis.
